# Non-coding RNA detection methods combined to improve usability, reproducibility and precision

**DOI:** 10.1186/1471-2105-11-491

**Published:** 2010-09-29

**Authors:** Peter Raasch, Ulf Schmitz, Nadja Patenge, Julio Vera, Bernd Kreikemeyer, Olaf Wolkenhauer

**Affiliations:** 1 Systems Biology and Bioinformatics Group, University of Rostock, D-18057 Rostock, Germany; 2 Department of Medical Microbiology, Virology and Hygiene, University Hospital Rostock, Schillingallee 70, D-18055 Rostock, Germany

## Abstract

**Background:**

Non-coding RNAs gain more attention as their diverse roles in many cellular processes are discovered. At the same time, the need for efficient computational prediction of ncRNAs increases with the pace of sequencing technology. Existing tools are based on various approaches and techniques, but none of them provides a reliable ncRNA detector yet. Consequently, a natural approach is to combine existing tools. Due to a lack of standard input and output formats combination and comparison of existing tools is difficult. Also, for genomic scans they often need to be incorporated in detection workflows using custom scripts, which decreases transparency and reproducibility.

**Results:**

We developed a Java-based framework to integrate existing tools and methods for ncRNA detection. This framework enables users to construct transparent detection workflows and to combine and compare different methods efficiently. We demonstrate the effectiveness of combining detection methods in case studies with the small genomes of *Escherichia coli*, *Listeria monocytogenes *and *Streptococcus pyogenes*. With the combined method, we gained 10% to 20% precision for sensitivities from 30% to 80%. Further, we investigated *Streptococcus pyogenes *for novel ncRNAs. Using multiple methods--integrated by our framework--we determined four highly probable candidates. We verified all four candidates experimentally using RT-PCR.

**Conclusions:**

We have created an extensible framework for practical, transparent and reproducible combination and comparison of ncRNA detection methods. We have proven the effectiveness of this approach in tests and by guiding experiments to find new ncRNAs. The software is freely available under the GNU General Public License (GPL), version 3 at http://www.sbi.uni-rostock.de/moses along with source code, screen shots, examples and tutorial material.

## Background

Non-coding RNAs have drawn much attention in the last couple of years, after being neglected for a long time [1]. They are now known to play key roles in diverse cellular processes such as regulation of gene expression, splicing and directing chemical modifications [2,3]. Functional categorization of RNAs is not yet complete as new functions are discovered continuously [4,5].

Detection of non-coding RNA genes in genomic sequences is an urgent but unsolved problem in bioinformatics [6]. The accelerated pace of sequencing technology further increases the need for reliable identification of ncRNAs [7]. The main approaches to computational prediction of ncRNAs are compositional analysis, secondary structure prediction, structural or sequence-based homology and the use of promoters and terminator signals. Numerous tools following one of these approaches or combinations thereof exist [6,8].

Compositional analysis can be a simple scan for local GC-content, an approach successful in AT-rich hyperthermophiles [9]. Considering more compositional features in a machine learning approach has also shown success [10]. Based on the fact that functional RNAs rely on a defined secondary structure, prediction of transcript minimum free energy is used as a means for detecting ncRNA genes [11]. Freyhult examined different quantities that can be used for this approach [12]. Sequence-based homology can be used for detection if reference genomes with appropriate evolutionary distances are available [13].

Successful tools such as QRNA [14] and RNAz [15] combine secondary structure prediction with a homology approach relying on multiple alignments. The most comprehensive RNA family database RFAM [16] uses covariance models combining structural and sequence conservation to establish RNA families. The covariance model can be used to find new members of existing families, however, at the expense of computational effort. Dynalign [17] uses an approximation of Sankoff's Algorithm for structural alignment of two RNAs.

Xiao *et al*. used promoter and terminator prediction in intergenic regions aided by conservation and secondary structure analysis to predict ncRNAs [18].

To achieve better accuracy, some tools limit the scope to specific ncRNA families such as tRNA, miRNA and snoRNA [6].

However, none of the available tools for *general *ncRNA detection has reached a level of reliability comparable to protein-gene detection software. In contrast to ncRNA genes, protein genes exhibit codon-bias, open reading frames and strong sequence conservation, simplifying their detection. Since the diverse methods for ncRNA detection are complementary, a practical approach is to combine the available methods, as suggested by recent reviews [6,8,19,20]. Meyer *et al*. also remarked that many ncRNA detection methods rest on the assumption of a significant secondary structure, which may not always be necessary for a ncRNA to function [8]. Consequently, even the more successful methods, which rely on this assumption, need to be complemented with others to achieve more comprehensive predictions.

The combination of methods allows for precise predictions by using candidates that are predicted by several methods, or finding more candidates by using predictions from all methods. If the combination is done under a well designed framework, reproducibility, transparency and comparison of predictions are improved as well.

Previous efforts for the integration of data and algorithms in genomic research exist: RNAStructure integrated secondary structure prediction and structure based homology analysis but is not easily extended and not readily useable for genomic scans [21]. Tools such as sRNAfinder [22] combine several approaches to improve prediction results, but in a predefined way. The UCSC genome browser offers a huge amount of experimental data, pre-calculated predictions and analyses for a selected number of genomes [23]. Basic functions for comparative genomics are available, extended by an interface to Galaxy. Galaxy is a project that also aims to overcome custom and redundant scripting for bioinformatics tasks in genomic research, but does not yet offer specialized tools for ncRNA prediction [24]. TAVERNA is a powerful all-purpose framework, but its primary source of functionality "BioCatalogue" does not yet contain essential ncRNA related tools such as RNAz and Dynalign [25]. LeARN is an extensible framework for annotating newly sequenced genomes, but it is more focused on processing trusted results from detection tools rather than improving predictions by the combination of analyses from different algorithms [26]. Consequently, there is a need for a framework that is easy to use and specialized for non-coding RNA detection. The main goals of our project are:

• **Combination: **Improving ncRNA detection by combining existing methods.

• **Comparison: **Easy comparison of the prediction performance of different methods must be possible.

• **Reproducibility: **application, combination and comparison of methods must be performed in a reproducible and transparent way.

• **Usability: **User experience should be improved by a GUI and visualization of all workflow steps and their respective results. No programming should be required to construct workflows, and to combine and compare methods.

Our software is aimed at three user groups: First, for bioinformaticians, the use and the combination of integrated tools must be simple. Second, developers of new algorithms for ncRNA detection must be provided with a ready-to-use environment and test bed. This removes the need to re-program solutions for tasks such as parsing files or visualization. Third, biologists must be able to re-use tested methods easily.

The implementation presented here supports compositional analysis, sequence-based homology (BLAST [27]), sequence and structural homology (RNAz [15] and Dynalign [17]) and secondary structure prediction (using RNAfold [28]). Our tool can easily be extended through an open architecture.

We will show how *moses *was designed to fulfill the given goals in the next section. In case studies we then demonstrate the effectiveness of combining methods: Precision or sensitivity are increased alternatively. Furthermore, our framework has been successfully applied to guide experiments in *Streptococcus pyogenes *to find new ncRNAs.

## Methods

### Key Idea

**Figure 1:**
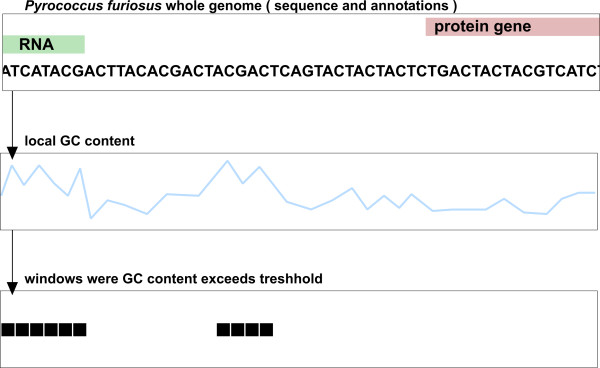
**Example detection workflow in *moses***. This simple detection workflow consists of three modules: the first one loads the sequence and annotations from a Genbank file, the second calculates the local GC-content in sliding windows, the third highlights windows, where the GC-content exceeds a certain threshold. In genomes of AT-rich hyperthermophiles, for instance, this simple method allows accurate prediction of ncRNAs.

**Figure 2:**
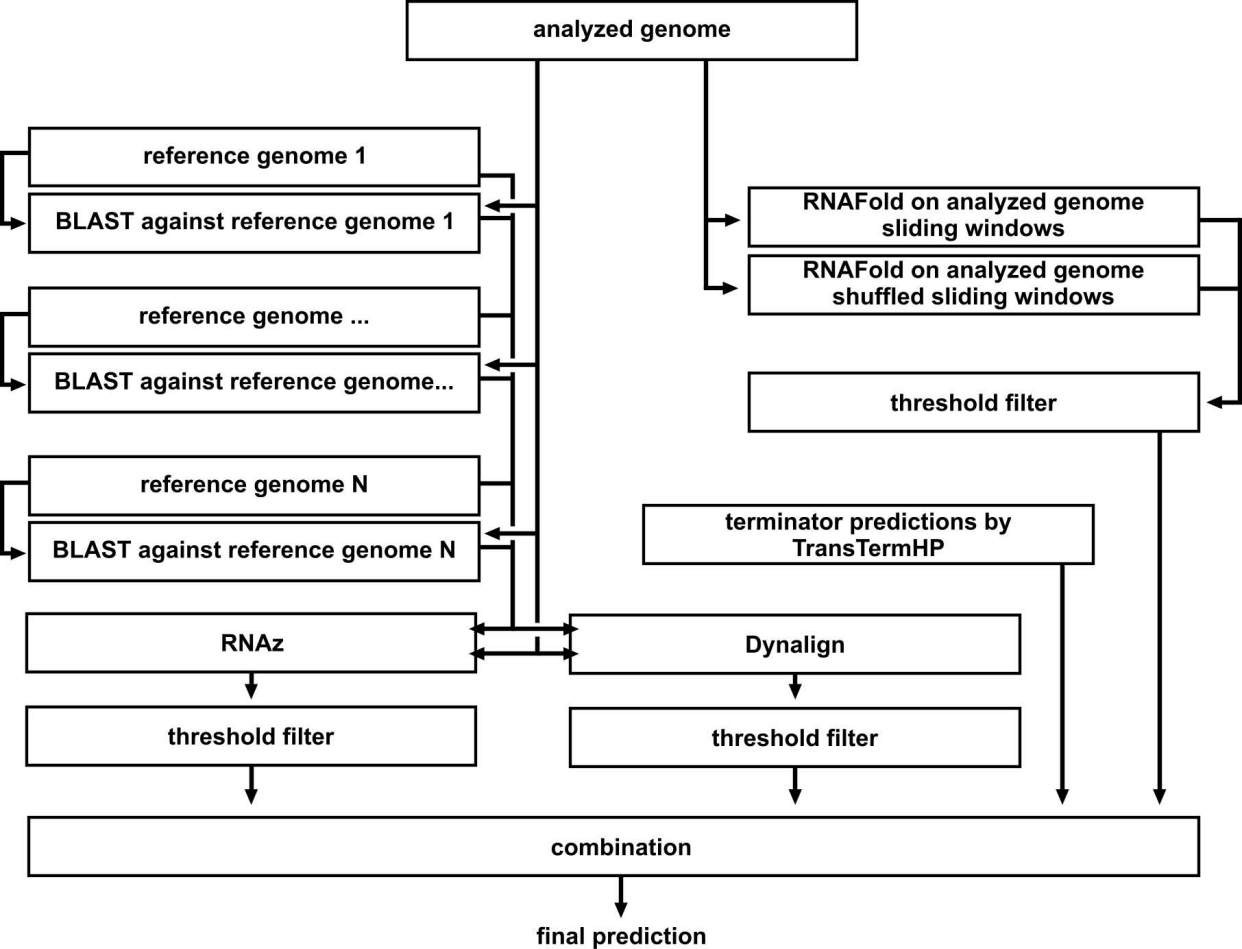
**Workflow used for tests of the combined detection method**. ***RNAz branch: ***The analysed genome is compared with each of the reference genomes using BLAST. It is then processed using a sliding window. For each window the best matching subsequences from the reference genomes, as found by BLAST, are retrieved. These subsequences and the sequence from the window of the analysed genome are then aligned by ClustalW (which is part of the RNAz module). Finally, the resulting alignment is analysed for conserved secondary structures by RNAz. If the prediction score given by RNAz is above the threshold of 0.9, the window is predicted to contain ncRNA. ***Dynalign branch: ***The analysed genome is processed using a sliding window, for each window the most similar regions of the reference genomes are given by the BLAST modules. The region with the highest BLAST score is used for structural alignment using Dynalign. The resulting alignment score is compared with a threshold to give a final prediction. ***RNAfold branch: ***The first RNAfold module predicts the minimum free energy of the minimum free energy structure for sliding windows of the analysed genome. Another RNAfold module does the same for shuffled versions of these windows. The obtained mean and standard deviation of the distribution are used to calculate a threshold: If the minimum free energy value of the original window is 3.5 standard deviations above the mean, the window is considered to contain a potential transcript with a highly defined secondary structure, an indication of a possible ncRNA. **Combination: **To combine the signals from the two methods, the overlap or the union of two or three methods is calculated to improve precision or sensitivity, respectively.

Our software *moses *(**mo**dular **se**quence **s**uite) processes and combines the results of different methods to find regions in a genome that contain ncRNA gene candidates. To do so, the user constructs a workflow from modules. Figure 1 shows a simple example of such a workflow. It consists of three modules: the first one loads a sequence from a Genbank file [29], the second calculates the GC-content using a sliding window for that sequence and finally a threshold filter module highlights regions of increased GC-content. In special cases such as *Pyrococcus furiosus*, increased GC-content is an accurate indication of a ncRNA gene [9]. After the threshold filter has been applied, for each window there is a prediction whether it contains a potential ncRNA gene or not. From this information a list of candidate locations can be constructed and used in experiments to verify the predictions. A more sophisticated detection workflow is shown in Figure 2. This workflow is also the one used for the case studies, described below.

An advantage of our modular approach is that it provides a good trade-off between flexibility and complexity: The user constructs workflows simply by chaining modules together and providing the parameters needed for its calculation. The output of every module can serve as the input for every other module. This allows for a free combination of modules while not requiring any programming skills.

The modules used to construct workflows can contain external tools, directly implemented analysis methods or helper functions. Each module represents one step in an analysis workflow. In the case of external tools, the module converts the input data, runs the tool, parses the output and converts it back into the *moses *format to ensure compatibility between all modules. Converting to one common data exchange format is more efficient than converting input and output between different tools, even though this is common practice in bioinformatics using custom scripts. The format we chose is a matrix of float values. Columns in the matrix correspond to nucleotide positions. Rows can hold different kinds of information, for instance ncRNA probability scores from several detection methods.

This basic format is very simple and yet can hold all types of information needed for the purpose of ncRNA prediction. The modules can be written using a data structure that is familiar to most programmers.

The parameters needed to run a module are saved in human readable format in the corresponding *moses*-file along with IDs identifying the source modules. This creates a structure of dependent calculations that form a detection workflow. Individual modules of this workflow can be exchanged to modify and re-use the workflow. For example, the modules holding the analysed genome can be exchanged to perform an identical scan on a different species.

### Main detection methods

The key modules *moses *provides are BLAST [27], word frequency analysis (typically used for GC-content analysis), RNAfold [28], RNAz [15] (using ClustalW [30] for alignments), Dynalign [17] and calculation of DNA properties, such as base stacking energy or bendability. BLAST can be used to compare two genomes, scan a genome for occurrences of a query sequence or locate conserved regions of a genome by BLASTing against a local database created with the BLAST helper tool formatDB. The RNAfold module uses a sliding window approach. For each sliding window the minimum free energy structure is predicted, and the corresponding minimum free energy value is stored at the centre position of the respective window. This results in a numerical profile aligned with the genome's base pairs.

The RNAz module scans a genome for ncRNA, requiring the output of several BLAST modules. Again, a sliding window approach is used. For each window, the most similar regions in reference genomes, as detected by BLAST, are used to construct a multiple alignment using ClustalW. This alignment is then analysed for ncRNA by RNAz. Finally the RNAz-score (called "RNA class probability") is stored at the window's centre position.

Similar to the RNAz module, the Dynalign module uses a sliding window and relies on BLAST modules to find the most similar regions in reference genomes for each window. A structural alignment of the analysed window and the region with the best BLAST-score is calculated using Dynalign. The output for each window is the alignment score.

The DNA properties module is similar to the RNAfold module, it calculates a numerical profile corresponding to a certain physical property of a DNA subsequence. The work of Abeel *et al*. shows that profiles of thermodynamic DNA properties can be used to detect transcriptional signals. Those signals not pointing to known protein genes may be indications of ncRNA genes [31]. DNA base stacking energy, bendability or protein induced deformability are examples for such properties. To calculate a numerical profile we use the procedure given by Abeel *et al*. [31]: Each (overlapping) dinucleotide of a DNA sequence is converted into a number according to a conversion table. These values are then smoothed using a sliding window, for each window the average is calculated and stored at the centre position of the window. Parameters for the properties were taken from EP3, a promoter detection tool developed by Abeel *et al*. [31]. The full list of available properties can be viewed on the tool's website (http://bioinformatics.psb.ugent.be/webtools/ep3/?conversion.)

Besides the key modules, predictions from RFAM can be incorporated by BLASTing against a RFAM dump. To include terminator predictions, output from TransTermHP [32] can be loaded as well.

All available methods can also be applied to sub-regions of a sequence. This is useful, for instance, to exclude protein gene regions from analysis or limit the calculations to a region of interest. Furthermore, *moses *provides a number of arithmetic and logical function to process the result of any method.

These functions are also applied for combining the detection methods to arrive at more precise or more sensitive predictions. Usually, this involves trading precision for sensitivity or vice versa. A more precise combined prediction is achieved by considering only predictions made by more than one individual method. A more sensitive combined prediction is achieved by collecting the predictions from all individual methods. A more sophisticated combination is to use weighted scores of individual methods: Reliable methods can be weighed higher then relatively unreliable ones to get a combined result that is more precise yet retains much of the sensitivity of the individual methods.

The quality of any single or combined detection method can be analysed and compared using the built-in statistical evaluation, if data of known ncRNAs is available.

### Graphical User Interface and Visualization

To make our software accessible to a wide range of users and to enhance usability, we provide a graphical user interface. Included features of the interface are:

• easy access to external tools as *moses *modules,

• constructing workflows with visualization of the modules' dependencies,

• multiple modes visualization of numerical profiles and for visual inspection, comparison and detection of correlations,

• browsing of genome annotations or calculated prediction signals,

• statistical assessment of each method, e.g., precision, sensitivity.

Integrated visualization of all intermediate results of a workflow helps finding mistakes, hypothesis generation and interpretation of results in the context of all available information.

## Results & Discussion

### Case Studies

To prove the effectiveness of combining detection methods, we used three methods, based on RNAz, Dynalign, RNAfold, and precalculated terminator predictions by TranstermHP on sets of known ncRNAs in *Escherichia coli*, *Listeria monocytogenes *and *Streptococcus pyogenes*. References for the known ncRNA sets are provided in Table 1. To give an reasonable estimation of the quality of the used methods we constructed test regions around known ncRNA extending 1500 bp up- and downstream around the known ncRNA. The flanking sequences serve as negative samples, the size was chosen to obtain a large number of negative samples while keeping the computational cost manageable. This results in regions composed of about 6% known ncRNA, 80% protein genes and 14% intergenic background, see Table 1. These compositions allow for the sampling of known positives as well as known negative regions in a realistic setting without the use of randomly generated negatives.

**Table 1:** Construction of the test regions for the case studies

	*Escherichia coli *str. K-12 substr. MG1655	*Listeria monocytogenes *EGD-e	*Streptococcus pyogenes *MGAS5005
regions size	222873 bp	284982 bp	212215 bp

known ncRNA	151 consisting of 11900 bp (5.3%) [29,35]	101 consisting of 19440 bp (6.8%) [36]	73 consisting of 14625 bp (6.9%) [34]

intergenic background	32754 bp (14.7%)	37317 bp (13.1%)	37067 bp (17.5%)

protein genes	180505 bp (81.0%)	231107 bp (81.1%)	163416 bp (77,0%)

On this test region, we used the workflow shown in Figure 2. The complete workflow with parameters and all data calculated for the three genomes is available on the *moses *website (http://www.sbi.uni-rostock.de/moses/data.html). The workflow consists of four branches, one for each of the RNAz, RNAfold and Dynalign based methods and one for the terminator predictions by TranstermHP. The first three methods use a sliding window with window size 75 sliding 1 base pair at a time. We checked for the influence of the window size by also trying the sizes 51 and 101 with the RNAz and the RNAfold method, see Table 2 for a comparison. The tests suggest that each method has a different optimal window size. Since we want to combine methods we chose the same window size for all methods. We chose 75 bp because it seems to produce almost as good results as 101 bp but at lower computational costs. Data for these tests is also provided on the *moses *website.

**Table 2:** Influence of the window size in *Escherichia coli*

method	RNAz			RNAfold		
window size	51	75	101	51	75	101

precision	0.21923937	0.21484879	0.23922414	0.2338333	0.2634066	0.24783753

sensitivity	0.23058824	0.12596639	0.13058823	0.07991596	0.10773109	0.1107563

Test of the RNAfold method (Z-Score threshold 3.5) and the RNAz method (threshold 0) in *Escherichia coli *for different window sizes.

The RNAz and the Dynalign branch both rely on BLAST modules that report for each window of the analysed sequence the most similar regions in four reference genomes. The reference regions are chosen to be the same size as the sliding windows. The reference genomes used are given in Table 3.

**Table 3:** Used Genomes

Species	Accession Number
**Tested Genomes**	

*Escherichia coli *str. K-12 substr. MG1655	NC_000913

*Listeria monocytogenes *EGD-e	NC_003210

*Streptococcus pyogenes *MGAS5005	NC_007297

**Reference Genomes**	

*Enterobacter *sp. 638	NC_009436

*Erwinia_tasmaniensis*	NC_010694

*Klebsiella pneumoniae *342	NC_011283

*Salmonella enterica *subsp. *enterica serovar Enteritidis *str. P125109	NC_011294

*Listeria innocua *Clip11262	NC_003212

*Listeria welshimeri serovar *6b str. SLCC5334	NC_008555

*Listeria seeligeri serovar *1/2b str. SLCC3954	NC_013891

*Streptococcus agalactiae *2603VR	NC_004116

*Streptococcus equi *subsp. *zooepidemicus *str. MGCS10565	NC_011134

*Streptococcus pyogenes *M1 GAS	NC_002737

*Streptococcus pyogenes *MGAS315	NC_004070

**Genome for new predictions**	

*Streptococcus pyogenes *NZ131	NC_011375

Genomes for BLAST sequence conservation analysis	see *moses *website

The RNAz module scans a genome for ncRNA using a sliding window. For each window, the most similar regions in the reference genomes, as detected by the BLAST modules, are alignment together with the analysed window using ClustalW. The resulting multiple alignment is then analysed by RNAz to give a so called "RNA class probability".

In the Dynalign method, only the reference region with the highest BLAST score is used for structural alignment using Dynalign.

The RNAfold method consists of two steps: First the minimum free energy value of the energetically optimal fold is calculated for each window. Second, the distribution of minimum free energy values for sequences of the nucleotide composition and length given by the analysed window is sampled. To this end RNAfold calculates the minimum free energy value for shuffled versions of the original window. The shuffling method by Altschul *et al*. [33] is used to preserve not only the mono - but also the dinucleotide composition, because the secondary structure prediction is especially sensitive to the dinucleotide composition. For our tests we used 100 shuffled versions. Mean and standard deviation are obtained from the sampled distribution to estimate the significance of the actual minimum free energy value. The final output of the RNAfold method is the Z-score for each window. The Z-score is the difference of the value of the original window and the mean in standard deviations. The RNAfold module inverts the sign of the minimum free energy values for convenience. The RNAfold method and the RNAz method are closely related but RNAz does not use the Z-Score of the original sequence, it rather uses averages of the Z-Scores from all sequences in the alignment. Our results show that the pure Z-Score as used by Kavanaugh *et al*. [11] is useful for ncRNA prediction, however, the way RNAz approximates Z-Scores is orders of magnitudes faster and practically of the same accuracy.

For the integrated tools BLAST, ClustalW and RNAz default parameters are used. Graphical output of predicted structures is suppressed for RNAfold to save computation time. TranstermHP predictions for *Listeria monocytogenes *and *Streptococcus pyogenes *were download from the TranstermHP website (http://transterm.cbcb.umd.edu/), predictions for *Escherichia coli *were performed using the downloaded program using default parameters.

The methods were combined by applying threshold filters to results of the RNAz, RNAfold and Dynalign method. The thresholds were 0.995, 4.5 and 550 respectively. For TranstermHP the confidence score threshold was 70, the default value used for the pre-calculated predictions from the TranstermHP website. Based on the parameter scans that were performed we selected values that gave intermediate precision and sensitivity for the individual methods.

After the thresholds have been applied in the centres of each window a "0" is stored if the value was below or equal to the threshold, "1" if above. The values for those three methods were added, additionally a "1" was added for each base pair of a predicted terminator.

**Figure 3:**
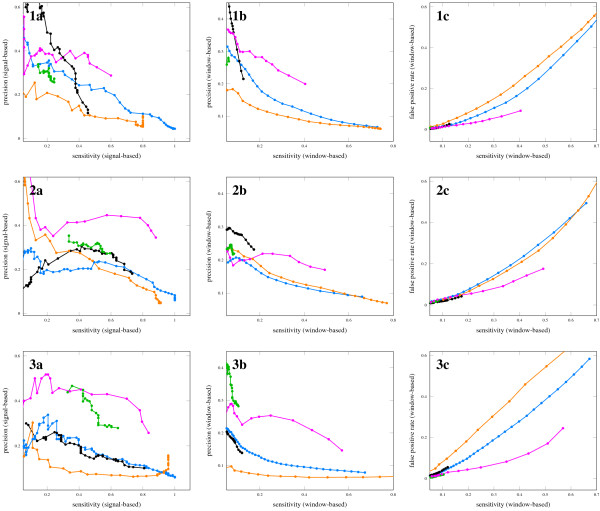
**Prediction performance of five methods in *Escherichia coli*, *Listeria monocytogenes *and *Streptococcus pyogenes***. Species 1a-c are results for *Escherichia coli*, 2a-c for *Listeria monocytogenes*, 3a-c for *Streptococcus pyogenes*. Blue graph: RNAfold-Zscore, threshold varied from 0 to 8.2 in steps of 0.2. Black graph: RNAz, probability score threshold varied from 0 to 0.9 in steps of 0.1, 0.9 to 0.99 in steps of 0.01, 0.99 to 0.999 in steps of 0.001, 0.999 to 0.9999 in steps of 0.0001, 0.9999 to 0.99999 in steps of 0.00001. Orange graph: Dynalign, alignment score threshold varied from 0 to 700 in steps of 20. Green graph: TranstermHP, confidence score varied from 70 to 100. Pink graph: combined method, score threshold varied from 0 to 1.5 in steps of 0.05.

The resulting sequence of integer values from 0-4 were then scanned using another sliding window. We tried different window sizes and 75 bp gave the best results (data available on the *moses *website). For each of those sliding windows the mean was calculated and another threshold filter was applied. We used thresholds from 0-1.5 in steps of 0.05, resulting in predictions with sensitivity and precision given in Figure 3.

Sensitivity and precision were calculated using the usual definitions: Sensitivity is the ratio of true positive windows to all known ncRNA-containing windows. Precision is the ratio of true positive windows to the sum of true positive and false positive windows.

While window-based precision and sensitivity are good to compare different methods, they do not reflect the practical value of predictions that are to be used to guide experimental verification of candidates. In practice, several windows next to each other that are predicted to contain ncRNA will be seen as one predicted locus or signal (for our purposes we want to neglect gaps). Those signals will then be used to guide experiments instead of each individual window.

Therefore, we define *signal precision *as the ratio of signals that overlap known ncRNA to all signals as an analogue to precision, and we define *signal sensitivity *as the ratio of known ncRNA that overlap a signal to all known ncRNA as analogue to sensitivity. The signal-based figures can be misleading if used alone, as too long signals will yield high signal precision and signal sensitivity without being specific enough for experiments. In order to check the quality of the predictions, we also calculated the false positive rate defined as the ratio of false positive windows to all windows known to not contain ncRNA. Figure 3 shows the prediction quality of the four individual methods and the combined method for all species in terms of window-based and signal-based figures as well as the false positive rates.

The plots reveal that for a wide sensitivity range the combined method largely improves the "signal-precision" by 15% and the window-based precision by about 10% across the three tested species. The improvements are confirmed by the reduced false positive rate visible in Figure 3, subfigures 3a-c.

Our tests show that our software allows for a flexible and easy combination of ncRNA detection methods and that the combination improves detection results. Methods can easily be compared using the available statistics.

### Prediction of novel ncRNAs

To show that *moses *can successfully guide experimental detection of ncRNA we present predictions for *Streptococcus pyogenes *NZ131, a human pathogen. We used four methods in a genomic scan to minimize false positives. As we have seen in the case studies, which were based on automated workflows, even the improved methods suffer from relatively high false positive rates. To arrive at a candidate list that had the most potential to be true ncRNA genes--in order to minimize unsuccessful experiments--we used manual inspection of multiple data sources instead.

All data calculated and the used parameters are available on the *moses *website.

The data sources were RNAfold secondary structure predictions, calculated DNA base stacking energy, BLAST-calculated conservation against related genomes and RNAz-predictions. The RNAfold module was used with window size 41, the DNA properties module with window size 81, step size 1 base pair in both cases. For RNAz the window size was 41 with step size 5 base pairs. The calculations were performed on the full genome sequence.

We examined the characteristic RNAfold and DNA base stacking energy profiles around known ncRNA genes to manually distinguish them from genomic background.

Also, isolated conserved spots were considered as clues for potential genes. Conservation was determining by BLASTing the NZ131 genome against all pyogenes serotypes in one module and against a selection of Streptococcus genomes in a second. Intergenic regions were examined for these four clues. Data used for the visual inspection is available on the *moses *website.

The procedure resulted in a list of 20 candidates. The features our selection of 20 candidates was based on, is listed in Table 4. We confirmed this list using the RNAz module with the same reference genomes as in the *Streptococcus pyogenes *test.

**Table 4:** Criteria for visual inspection of intergenic regions in *Streptococcus pyogenes NZ311*

	start	1) BLAST vs *Streptococci*	2) BLAST vs *S. pyogenes*	3) DNA base stacking energy	4) RNAfold
1	202790	X		X	

2	216807	X		X	X

3	273446	X		X	X

4	308253		X	X	X

5	349593		X	X	X

6	362796		X		X

7	363720	X	X		X

8	407642		X	X	

9	506298	X	X		X

10	512500	X	X		X

11	554314	X		X	X

12	568980	X	X	X	

13	806825		X		X

14	827250	X	X		

15	949486	X			X

16	1201476		X		X

17	1487751	X	X	X	

18	1571435		X		X

19	1581266		X		

20	1704825	X		X	X

Criteria: 1) increased conservation shown by BLAST against selected *Streptococci *genomes (see the *moses *website for a List). 2) increased conservation shown by BLAST against all *Streptococcus pyogenes *substrains. 3) increased DNA base stacking energy profile or presence of two nearby peaks. 4) a peak in the RNAfold profile.

**Figure 4:**
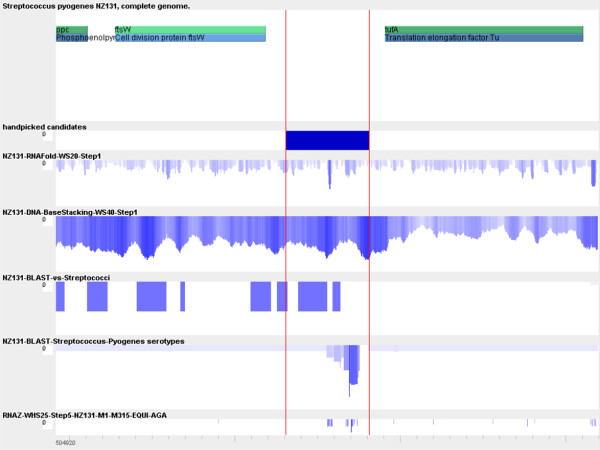
**Data used for determination of ncRNA candidates in *Streptococcus pyogenes***. This screenshot shows an example of the data used to find intergenic regions that may contain ncRNA genes. The first row contains annotations from the Genbank file downloaded from NCBI. Green and blue bars correspond to protein genes. The second row contains the hand-picked candidate regions. The blue block delimits the region where an ncRNA is suspected. Start and end of this region are not accurate as the methods used do not allow for exact determination of the transcript. The red lines indicate the start and end of the candidate regions for visual orientation. The third row contains the RNAfold minimum free energy profile, a clear peak is visible indicating a strong RNA structure. Row four contains the DNA base stacking energy profile, two peaks delimit the candidate region and a third peak is inside, from our visual inspection of all intergenic regions this profile is notably different. Row five and six contain the scores for each window of the analysed genome for a BLAST search against all Streptococcus genomes and against all *Streptococcus pyogenes *genomes respectively, notable are the conserved regions *outside *the protein genes. The last row contains the RNAz predictions. The shades of blue in lines 2-7 correspond to the height of the respective bars. Screenshots for all four candidates are given on the *moses *website.

From the 20 candidates, four highly probable candidates were selected based on RNAz prediction and presence of a putative terminator, visible as a peak in the RNAfold profile. As the minimum free energy value calculated by RNAfold is a measure of the thermodynamic stability of RNA structures, such a peak can be an indicator of ncRNAs as well as of terminators. The locations of the putative terminators were used to aid placement of the downstream primer for the RT-PCR experiments, as a terminator gives an indication of where the 3' end of a possible ncRNA is located. RNAz predictions were used as the most probable centre of the potential transcript. An example of the information calculated in *moses *around the candidate regions is displayed in Figure 4. Corresponding screenshots of all four candidate regions and data used for primer design with genomic coordinates are given on the *moses *website.

The combination of multiple methods, possible in *moses*, has yielded highly probable candidates. RNAz or BLAST alone, for instance, would have given us hundreds of candidate loci to examine (data on the *moses *website).

**Figure 5:**
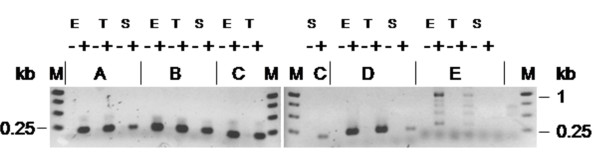
**Presence of predicted ncRNA transcripts in cDNA of *Streptococcus pyogenes *M49 grown to different phases of the growth cycle**. Analysis of RT-PCR products by agarose gel electrophoresis. Details of the experimental procedure are given on the *moses *website. M: molecular weight marker, A-D: mopsRNA1-4 (candidate genes); E: M protein encoding emm gene (control), (-) RT mock reactions, (+) RT reactions containing reverse transcriptase, E: exponential growth phase, T: transition growth phase, S: stationary growth phase.

Expression of the four highly probable candidates was verified by reverse transcription (RT) followed by PCR, see Figure 5. Reactions without addition of the RT enzyme served as negative controls. Reverse transcription of the EMM-gene was performed as a positive control, which is known to be expressed in this strain under the conditions tested in our experiments. Details of the experimental procedure, including the used primers, are available on the *moses *website and in Additional file 1. The candidates were named mopsRNA1-4 (***mo**ses ***p**redicted **s**mall RNA). Candidates 1 and 4 overlap with candidates reported as predictions by a previous bioinformatic search in the study of Perez *et. al *[34], labelled SR307231, SR759205 and SR758876.

Our predictions demonstrate that the integrated approach possible with *moses *is able to guide experimental detection of new ncRNAs. The RT-PCR experiments are not sufficient to rule out that the observations are related to neighbouring transcripts rather than true ncRNA. Accordingly, further experiments are in progress to confirm and characterize the four new candidate ncRNAs and their function in *Streptococcus pyogenes *physiology and virulence.

## Limitations

The computationally more demanding algorithms RNAz-analysis and RNAfold secondary structure prediction with shuffled comparison sequences need approximately 120 hours for full prokaryotic genomes (assuming an average size of 4 MB) on standard workstation computers. Dynalign takes even longer because it performs full structural alignments.

Our tests were performed on a machine with Intel(R) Core(TM) 2 Duo CPU 2.66 GHz with 2 GB of RAM with the use of parallelization. The RNAfold method and then RNAz method both took three hours for 99.950 analysed windows (for an analysed genome, the number of base pairs minus the window size plus one equals the number of windows to analyse). The window size for both methods was 51. The windows step was one base pair to obtain maximum resolution. However, this is not to imply that we can predict the exact gene starts and ends. For the RNAfold method 100 shuffled windows were used and three reference genomes for the RNAz method. If the analysis is limited to the intergenic regions, the time is reduced depending on the percentage of coding regions of the genomes under examination (often, intergenic regions constitute 10% of prokaryotic genomes). Other ways to avoid too long calculations include choosing a larger step size and parallel calculations by dividing analysed genomes in smaller parts.

The methods used here are in principle not restricted to prokaryotes, but to the sheer size of the genome.

## Conclusions

We developed a framework for reproducible, transparent and easy combination of existing ncRNA detection methods. Our contribution helps to satisfy the need for a combined approach as suggested by recent reviews [6,8,19,20]. The main improvements our framework provides are:

### Improved ncRNA detection by combining existing methods

Wrapping existing tools and methods in *moses *modules that convert input and output formats to a common data interchange format makes combination possible. We have demonstrated the effectiveness of combining methods in tests on *Escherichia coli *and *Streptococcus pyogenes*. Further we predicted novel ncRNAs in *Streptococcus pyogenes *using multiple methods to yield highly probable candidates, thereby reducing unsuccessful experiments. Final confirmation and subsequent characterisation of the candidates is in progress.

### Facilitated comparison of methods

The used methods can readily be compared using the integrated accuracy report. Statistical figures such as signal based and by-base-pair precision, sensitivity are readily at hand. This allows effective evaluation of existing methods and the selection of appropriate methods, e.g., according to available reference genomes or given taxon.

### Improved reproducibility, re-usability and transparency

Workflows are self-documented as all parameters and data dependencies are stored in the *moses *files. This means the workflows are transparent because no hidden conversions and no implied functions are performed, only the ones defined by the user. No custom scripts or custom in-house software is involved in studies carried out in *moses*. Furthermore, an existing workflow can be reused on different sequences, different data or altered parameters.

### Improved Usability

We created a GUI and visualization for all intermediate steps of a workflow. This enables to detect flaws in a workflow and helps to interpret the results. The integrated environment supports hypothesis generation and brings data and results in context with all available information.

The method of constructing workflows in *moses *is easy as it requires no programming and no scripting. This makes it an attractive tool for bioinformaticians. Extending the framework with new algorithms is made easy through an open architecture with a plug-in mechanism. Programming effort is thus minimized and developers of new algorithms are provided with a ready-to-use platform. Biologists can easily reuse existing workflows.

### Outlook

The next step in the development of our framework is the integration of further existing methods and algorithms. Combination of methods could be enhanced by including support for SVM training and classification. Possibly, in the course of adding more tools the scope could be expanded to not only find ncRNA genes but protein genes, promoters, terminators and transcription factor binding sites as well. The result would be a complete picture of a genome under one common framework.

A recent approach to detect regulatory regions is pattern recognition in profiles of physical properties of the DNA, see for instance [31]. As our framework offers different sources for such profiles, not only based on physical properties, it is a natural extension of our work to apply pattern detection to the profiles calculated by *moses*.

## Authors' contributions

PR and US designed the software framework. PR implemented the framework, performed the tests and predicted the new ncRNAs. BK and NP carried out the RT-PCR experiments to verify the predicted ncRNAs. OW participated in the design of the framework and helped to draft the manuscript. All authors read and approved the final manuscript.

## Supplementary Material

Additional file 1 **Experimental procedure for verification of four selected ncRNA candidates**. Additional file 1 is a Microsoft Word document with a detailed description of the materials and methods employed for experimental verification of the final four ncRNA candidates. Click here for file
