# Supplementary material for: Non-coding RNA detection methods combined to improve usability, reproducibility and precision
Source: BMC Bioinformatics. 2010 Sep 29;11:491. doi: 10.1186/1471-2105-11-491 (PMC2955705; doi:10.1186/1471-2105-11-491)
Supplement: Additional file 1 — Experimental procedure for verification of four selected ncRNA candidates. Additional file 1 is a Microsoft Word document with a detailed description of the materials and methods employed for experimental verification of the final four ncRNA candidates. [file 1471-2105-11-491-S1.DOC]

**Material and Methods**

***Bacterial strains and culture conditions***

A serotype M49 strain of *Streptococcus pyogenes* was used to isolate RNA and chromosomal DNA. This strain (591) is a clinical isolate from a skin infection and was kindly provided by R. Lüttiken (Aachen). Bacteria were grown in Todd-Hewitt broth (Invitrogen) supplemented with 0,5% yeast extract (Invitrogen) (THY medium) at 37°C under a 5% CO_2_-20%O_2_ atmosphere.

***DNA, RNA, and cDNA techniques***

Purification of chromosomal DNA from *Streptococcus pyogenes* was performed as outlined by Kreikemeyer et al. (2001). Total bacterial RNA from cultures grown to exponential, transition and stationary phase of growth, respectively, was isolated using the FastRNA©ProBlue Kit from MP Biomedicals as outlined in the protocol provided by the manufacturer. As an additional step the purified total RNA was digested with DNase to remove remaining traces of chromosomal DNA. For this, RNA was treated with 10U of DNase for 30 min at 37°C and after this digestion step the samples were incubated at 72°C for 5 min to inactivate the DNase. The purified total RNA was then reverse transcribed to generate cDNA using the First-Strand cDNA Synthesis Kit from Invitrogen following the protocol provided by the manufacturer. As negative controls served reactions without addition of the reverse transcriptase. cDNA products were amplified by PCR with primers specific for the respective candidate sequences. For PCR, the cDNA was mixed with 10µM of each forward and reverse primer in a total volume of 50µl, and after addition of PCR buffer containing 15 mM MgCl_2_ and 5U/µl Taq polymerase (Qiagen, Germany) the following PCR program was used to amplify the candidate sncRNA encoding genes: 95°C for 1 min, 24 cycles consisting of 30 s template denaturation at 95°C, 30 s of primer annealing at 55°C, and 1 min extension at 72°C, respectively. After 24 cycles a final extension of 5 min at 72°C was performed. 10µl of the PCR reactions were separated on a 2 % agarose gel in order to visualize the amplified products. As a positive control, one PCR with primers specific for the *emm* gene was performed, which is known to be expressed under the conditions investigated in this study. The following primers were designed based on the full genome sequence of *S. pyogenes* M49 strain NZ131 (NCBI accession number: NC011375) and were used for amplification of the suspected sncRNA candidate genes:

mopsRNA1-For: 5^´-^ctgaccccaaaaagttggac-3`

mopsRNA1-Rev: 5´-ggcctcaactagccaatcaa-3´

(expected product size: 232 bp);

mopsRNA2-For: 5´- gtgtgacatgagcccaaaca-3´

mopsRNA2-Rev: 5´- cggtaggccatacagtctaactt-3´

(expected product size: 217 bp);

mopsRNA3-For: 5´- gtgacgttacgcggaatttt-3´,

mopsRNA3-Rev: 5´- tcgatgacctctttgctcct-3´

(expected product size: 206 bp);

mopsRNA4-For: 5´- accgagagaactccgtttga-3´

mopsRNA4-Rev: 5´- gagccccaaaagttggctac-3´

expected product size: 248 bp).

Primers amplifying the M protein-encoding *emm* gene of *S. pyogenes* were used as positive controls in this RT-PCR. The all-*emm* primers were as follows:

all-M-For: 5´-ATAAGGAGCATAAAAATGGCT-3´,

all-M-Rev: 5´-AGCTAAGTTTTCTTCTTTGGG-3´

(expected product size: 1200 bp).
